# sFlt‐1/PlGF Ratio in the Diagnosis of Preeclampsia on the MAGLUMI X3 Analyzer

**DOI:** 10.1002/jcla.70302

**Published:** 2026-07-05

**Authors:** Pavel Broz, Simona Kukralova, Zdenek Sebesta, Katerina Brslicova, Michal Kozerovsky, Jaroslav Racek, Daniel Rajdl

**Affiliations:** ^1^ Institute of Clinical Biochemistry and Hematology University Hospital in Pilsen Pilsen Czech Republic; ^2^ Faculty of Medicine in Pilsen Charles University in Prague Pilsen Czech Republic; ^3^ Department of Obstetrics and Gynecology University Hospital in Pilsen Pilsen Czech Republic

**Keywords:** MAGLUMI X3, method comparison, PlGF, preeclampsia, sFlt‐1

## Abstract

**Objectives:**

To compare the analytical and clinical performance of MAGLUMI X3 (Snibe) and Cobas e602 (Roche) assays for sFlt‐1, PlGF, and the sFlt‐1/PlGF ratio in women hospitalized at the Department of Gynecology and Obstetrics, University Hospital in Pilsen, and to evaluate diagnostic accuracy for early‐ and late‐onset preeclampsia (PE).

**Methods:**

This single‐center real‐world study analyzed 192 serum samples from 164 patients (mean age 31.8 ± 5.0 years). Routine testing was performed on Cobas e602; residual serum was measured on MAGLUMI X3 within 24 h. Precision followed CLSI EP05‐A3/EP15‐A3; agreement used Passing–Bablok and Bland–Altman. Diagnostic performance was evaluated by ROC curves with AUC and 95% confidence intervals (CI); AUCs were compared by the DeLong test.

**Results:**

Repeatability and reproducibility were acceptable across levels (patient‐sample CVs ~0.7%–2.0% and 1.1%–1.5%; QC CVs ~2.0%–8.9%). Bland–Altman showed a mean bias for sFlt‐1 of −869.9 ng/L (95% CI −1035.6 to −704.2) and for PlGF +6.2 ng/L (−4.6 to 17.0). Spearman's r was 0.99 (sFlt‐1) and 0.97 (PlGF), *p* < 0.0001. For clinical classification, AUCs were high for both platforms—late‐onset PE: Roche 0.95 (95% CI 0.86–0.99) and Snibe 0.95 (0.87–0.99); early‐onset PE: Roche 0.85 (0.75–0.92) and Snibe 0.84 (0.75–0.91)—with no significant differences by DeLong (*p* = 0.71 late; *p* = 0.32 early).

**Conclusions:**

MAGLUMI X3 showed acceptable analytical performance and diagnostic discrimination comparable to Cobas e602. Although sFlt‐1 values were systematically higher on MAGLUMI, overall discrimination remained similar across platforms when platform‐specific cut‐offs were applied.

We compared the analytical and clinical performance of MAGLUMI X3 (Snibe) and Elecsys (Roche) for measuring sFlt‐1, PlGF, and their ratio in women hospitalized at the Department of Gynecology and Obstetrics, University Hospital in Pilsen. In a single‐center, real‐world cohort (192 samples from 164 patients), method comparison showed good agreement between analyzers. Diagnostic discrimination for preeclampsia (PE vs. non‐PE) was similar across platforms (AUC early‐onset 0.84–0.85; late‐onset 0.95 each; no significant difference by DeLong).

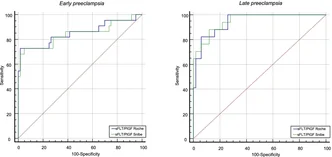

**Abbreviations**
AUC: area under the ROC curve
CI: Confidence interval
CLIA: chemiluminescence immunoassay
CV: Coefficient of variation
GDPR: General Data Protection Regulation
LoD: Limit of detection
LoQ: Limit of quantification
PE: Preeclampsia
PlGF: Placental growth factor
QC: Quality control
ROC: receiver operating characteristic
SD: Standard deviation
sFlt‐1: Soluble fms‐like tyrosine kinase‐1
SOP: standard operating procedure

## 1 Introduction

Preeclampsia (PE) is a pregnancy‐specific disorder diagnosed after 20 weeks of gestation, typically characterized by new‐onset hypertension and proteinuria and/or other signs of maternal organ dysfunction. In severe cases, it may progress to eclampsia, defined by the occurrence of seizures, or to HELLP syndrome (hemolysis, elevated liver enzymes, and low platelet count), the diagnosis of which relies predominantly on laboratory findings [1].

The exact pathophysiology of PE remains incompletely understood. Placental vascular dysfunction occurs with the subsequent release of agents affecting the overall maternal hemodynamics. Pro‐angiogenic and anti‐angiogenic factors play an important role in the development of PE. These are the pro‐angiogenic PlGF (placental growth factor), the anti‐angiogenic sFlt‐1 (soluble fms‐like tyrosine kinase‐1), and their sFlt‐1/PlGF ratio, the value of which is indicative of the risk of developing PE. These biomarkers can be detected in the serum of pregnant women before the manifestation of clinical signs of the disease [2]. Laboratory testing is becoming an increasingly important part of the diagnosis and prediction of PE. The primary goal of the present study was to compare the performance of the MAGLUMI X3 analyzer (Snibe) with the routinely used Cobas e602 analyzer (Roche) in measuring sFlt‐1 and PlGF concentrations. A secondary objective was to investigate whether any potential analytical differences between these methods could influence the clinical classification of patients based on the sFlt‐1 and PlGF ratio.

## 2 Materials and Methods

### 2.1 Patients and Clinical Data

During the study period, 164 patients (age 31.8 ± 5.0 years; data presented as mean ± SD) hospitalized at the Department of Gynecology and Obstetrics of the University Hospital in Pilsen were assessed; a total of 192 serum samples from 164 women were included in the analytical comparison. For clinical performance analysis, only cases with complete clinical follow‐up data were included, resulting in 149 evaluable patients. The study included patients where various diagnoses were present in some cases (presented as diagnosis–number of cases: Obesity–52, Diabetes/Gestational Diabetes Mellitus–46, Hypothyroidism–22, Fetal Growth Restriction–21, Group B Streptococcus positivity–12, Thrombophilia–5, Endometriosis–5, Celiac Disease–4, Chorioamnionitis–4, Nicotine Use–4, Uterus Myomatosus–3, Asthma–2, Rh Incompatibility–2, Thrombocytopenia–2, Autoimmune Thyroiditis–2, Latent Syphilis (Lues latens)–2, Pregnancy Anemia–1, Intrahepatic Cholestasis of Pregnancy–1); however, suspicion of PE in the differential diagnosis was present simultaneously. Blood samples were taken from the antecubital vein while the participant was in a seated position into a VACUETTE TUBE 4 mL Serum Separator (Greiner Bio‐One GmbH, Kremsmünster, Austria). No adverse events occurred related to sample collection or testing (venipuncture only).

### 2.2 Sample Collection and Handling

All samples were centrifuged upon arrival at the laboratory at 2,000 g for 10 min, as established for routine analyses. After the routine analysis (including the determination of sFlt‐1 and PlGF on the routine analyzer), residual serum with sufficient volume was stored at 2°C–8°C and analyzed on the MAGLUMI X3 analyzer within 24 h, in accordance with the stated stability of the analytes. Routine analysis of sFlt‐1 and PlGF concentrations was performed using the Elecsys sFlt‐1 and Elecsys PlGF kit on the Cobas analyzer–Cobas 8000, e602 module (Roche Diagnostics, Basel, Switzerland). Additional analysis was performed using MAGLUMI sFlt‐1 and MAGLUMI PlGF (Snibe Diagnostic, Shenzhen, China). No invalid or indeterminate results occurred; all runs met quality control (QC) acceptance criteria. Operators performing measurements on each platform were blinded to clinical outcomes and to results from the other platform.

### 2.3 The Assay Platforms

The tested MAGLUMI X3 analyzer is a fully automated analyzer for qualitative and/or quantitative analysis of analytes in human samples. The principle of MAGLUMI sFlt‐1 and MAGLUMI PlGF is a chemiluminescence sandwich immunoassay (CLIA) with magnetic microparticles. The required volume for MAGLUMI sFlt‐1 immunoassay is 30 μL, and for MAGLUMI PlGF is 50 μL. The shortest time from register time to finish time for these two methods is approximately 30 min. The limit of detection (LoD) for MAGLUMI sFlt‐1 is 10.0 ng/L, and the limit of quantitation (LoQ) is 15.0 ng/L (measuring range defined by the LoD and the maximum of the master curve 10.0–85,000.0 ng/L). For MAGLUMI PlGF, the LoD is 3.0 ng/L, and the LoQ is 9.0 ng/L (measuring range reported by the manufacturer defined by the LoD and the maximum of the master curve 3.0–10,000.0 ng/L).

Cobas e602 uses the principle of electrochemiluminescence sandwich immunoassay (ECLIA) for the sFlt‐1/PlGF ratio. The required volume for the sFlt‐1 immunoassay is 20 μL, and for PlGF is 50 μL. The total duration of these two assays is 18 min. The LoD of Elecsys sFlt‐1 is 10.0 ng/L, and the limit of quantitation is 15.0 ng/L (measuring range defined by the LoD and the maximum of the master curve 10.0–85,000.0 ng/L). For Elecsys PlGF, the LoD is 3.0 ng/L, and the LoQ is 10.0 ng/L (measuring range reported by the manufacturer, defined by the LoD and the maximum of the master curve 3.0–10,000.0 ng/L).

### 2.4 The Statistical Analysis and Clinical Evaluation

Normality of data distribution was assessed using the Kolmogorov–Smirnov test. Correlations were evaluated by Spearman's rank correlation. Agreement between assays was analyzed using Passing–Bablok regression and Bland–Altman plots. No data were missing for the primary analyses. The sFlt‐1/PlGF ratio was calculated from the results of both analyzers. The diagnosis of PE was retrospectively determined from the clinical information system at the time of patient discharge. Preeclampsia was defined as new‐onset hypertension (systolic blood pressure ≥ 140 mmHg or diastolic blood pressure ≥ 90 mmHg) after 20 weeks of gestation, accompanied by proteinuria (≥ 300 mg/24 h) and/or evidence of maternal organ dysfunction. This definition is consistent with current international recommendations of the International Society for the Study of Hypertension in Pregnancy (ISSHP), the American College of Obstetricians and Gynecologists (ACOG), and the National Institute for Health and Care Excellence (NICE) [3, 4, 5]. Patients were classified into PE and non‐PE groups. Diagnostic accuracy was assessed by receiver operating characteristic (ROC) curve analysis with calculation of the area under the curve (AUC) and 95% confidence intervals; AUCs were compared using the DeLong test. Cohen's kappa coefficient was used to assess agreement between the two laboratory systems in classifying PE using the sFlt‐1/PlGF ratio thresholds for early (< 34 weeks of gestation) and late PE (≥ 34 weeks of gestation). A *p*‐value < 0.05 was considered statistically significant. Statistical analyses were performed with MedCalc Statistical Software version 20.216 (MedCalc Software Ltd., Ostend, Belgium; https://www.medcalc.org; 2023). No formal sample size calculation was performed; all eligible cases within the study period were included.

### 2.5 Analytical Studies

The repeatability (within‐run precision) of both assays was determined using serum samples from patients at four concentration levels in accordance with CLSI EP05‐A3 and EP15‐A3 [6]. For each level, the mean, standard deviation (SD), and coefficient of variation (CV%) were calculated. Reproducibility (between‐run precision) was assessed from repeated measurements performed on different days. Manufacturer‐provided control material with assigned target values traceable to the assay's calibration system was used. Methods have been standardized against the Snibe internal reference standard. Bias was calculated as the difference between the mean measured concentration and the assigned/target value. Method comparison between the two analytical systems for both sFlt‐1 and PlGF was performed using Bland–Altman analysis to estimate bias and the 95% limits of agreement.

## 3 Results

A total of 192 serum samples from 164 women were included in the analytical comparison. For clinical performance analysis, 149 patients with complete clinical data were included. Basic analytical properties, including repeatability, bias, and related parameters, are stated in Tables 1 and Table 2. Repeatability was measured using serum samples from patients.

**TABLE 1:** Repeatability of sFlt‐1/PlGF on selected values measured using serum samples from patients, *n* = 10. data presented in ng/L.

	PlGF A	PlGF B	PlGF C	PlGF D	sFlt‐1 A	sFlt‐1 B	sFlt‐1 C	sFlt‐1 D
Average	25.8	754.4	123.7	25.7	10,039.6	1,410.8	19,942.9	10,702.3
Min	25.1	746.0	121.0	25.1	9,913.0	1,373.0	19,599.0	10,528.0
Max	26.8	764.0	126.0	26.2	10,200.0	1,425.0	20,130.0	10,871.0
SD	0.5	5.6	1.8	0.4	145.2	16.7	215.7	116.8
CV (%)	2.0	0.7	1.4	1.5	1.5	1.2	1.1	1.1

**TABLE 2:** Reproducibility and bias were determined using control material. Manufacturer‐provided control material with assigned target values traceable to the calibration system of the assay was used. The standard deviation was calculated, and the obtained values are presented in the table. The number of analyses was 45 for PlGF and 41 for sFlt‐1, respectively. Data presented in ng/L. QC–Quality control, SD–Standard deviation, CV–Coefficient of variation.

QC	PlGF	QC2	sFlt‐1	QC2
	QC1		QC1	
Target value (range)	200.0 (140.0–260.0)	1,200.0 (840.0–1,560.0)	400.0 (280.0–520.0)	3,000.0 (2,100.0–3,900.0)
Average min–max	176.7 (147.0–199.0)	1,143.4 (920.0–1,246.0)	413.7 (400.0–427.0)	3,227.2 (3,076.0–3,327.0)
SD	15.7	93.7	8.4	64.7
CV (%)	8.9	8.2	2.0	2.0
Bias	−11.7	−4.7	3.4	7.6
Uncertainty	17.8	16.4	4.1	4.0

**FIGURE 1:**
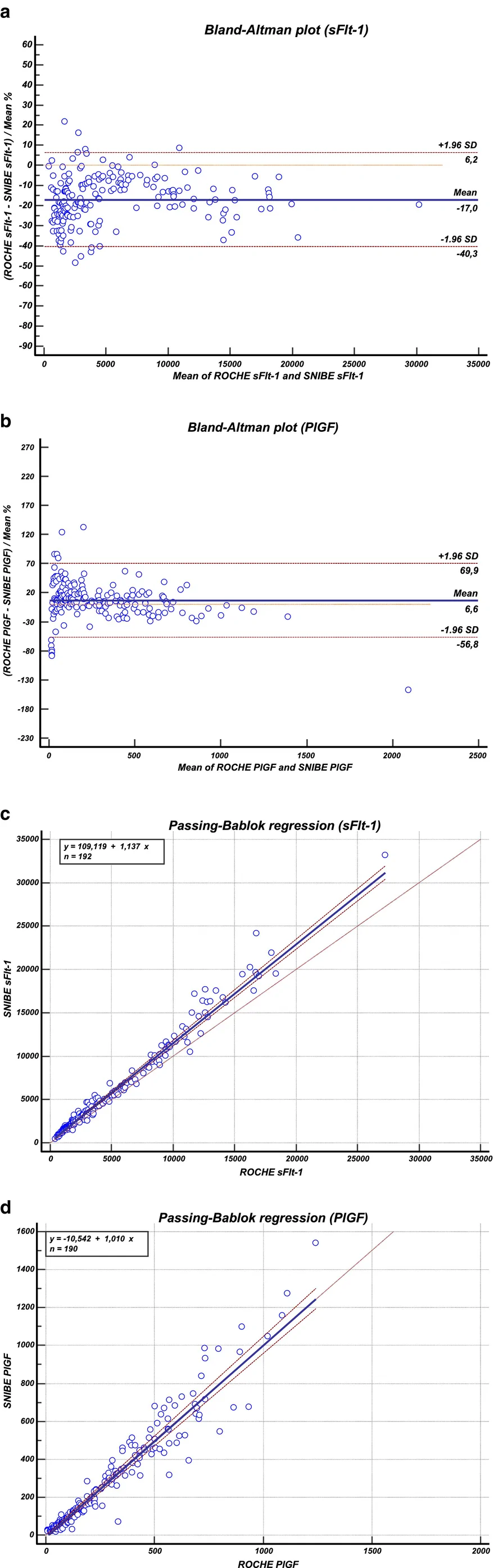
(a–d) Bland–Altman plots and Passing–Bablok regression analyses. Data visualized as % for better presentation of data. Regression equations and identity lines are included. Data presented in ng/L. Spearman's correlation coefficient for sFlt‐1 values was 0.991, and for PlGF values was 0.97, *p* < 0.0001 in both cases. sFlt‐1–Soluble fms‐like tyrosine kinase‐1, PlGF–placental growth factor.

Spearman's correlation coefficient for sFlt‐1 values was 0.99, and for PlGF values, 0.97, respectively. Bland–Altman plots and Passing–Bablok analysis are presented in Figure 1. The average bias (95% CI) for sFlt‐1 was −869.9 (−1,035.6 to −704.2) ng/L, *p* < 0.0001, and for PlGF, 6.2 (−4.6 to 17.0) ng/L, *p* = 0.26, indicating that the Roche method measured lower values compared to the Snibe method for sFlt‐1.

**FIGURE 2:**
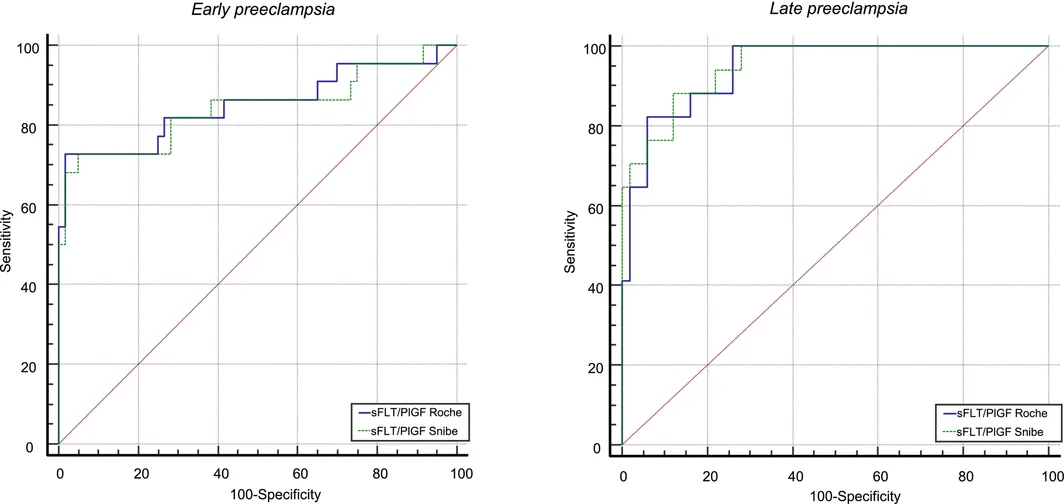
ROC curves for sFlt‐1/PlGF ratio in the diagnosis of early (left) and late (right) PE. ROC curves in both cases did not show statistically significant differences using the DeLong test, with *p* = 0.71 for late and *p* = 0.32 for early PE.

The ROC curves of early and late PE and corresponding results are presented in Figure 2. Among the subset with complete clinical data used for clinical analyses, early PE was diagnosed in 22 of 82 cases, whereas late PE was diagnosed in 17 of 67 cases. The DeLong test showed no significant differences (*p* = 0.71 for late and *p* = 0.32 for early PE). Values of Cohen's kappa were 0.97 for early and 0.89 for late PE. Data with sensitivity, specificity, and accuracy, including 95% CI, are presented in Table 3. For transparency, 2 × 2 cross‐tabulations at the proposed cut‐offs are provided in Table S1, together with sensitivity and specificity (95% CI).

**TABLE 3:** Proposed sFlt‐1/PlGF cut‐off values for selected laboratory methods, determined at points corresponding to the maximum area under the ROC curve (AUC) with adequate sensitivity and specificity. the table includes corresponding sensitivity, specificity, and accuracy with 95% confidence intervals. ROC–Receiver operating characteristic; CI–Confidence interval; PE–Preeclampsia.

Method and cut‐off value (ng/L)	Area under the ROC curve (95% CI)	Diagnostic sensitivity (95% CI)	Diagnostic specificity (95% CI)	Accuracy (95% CI)
sFlt‐1/PlGF Roche late PE (81.0)	0.95 (0.86–0.99)	82.4 (56.6–96.2)	94.0 (83.5–98.7)	0.891 (0.79–0.95)
sFlt‐1/PlGF Snibe late PE (89.0)	0.95 (0.87–0.99)	88.2 (63.6–98.5)	88.0 (75.7–95.5)	0.875 (0.77–0.94)
sFlt‐1/PlGF Roche early PE (38.5)	0.85 (0.75–0.92)	72.7 (49.8–89.3)	98.3 (91.1–100.0)	0.902 (0.82–0.95)
sFlt‐1/PlGF Snibe early PE (56.0)	0.84 (0.75–0.91)	72.7 (49.8–89.3)	95.0 (86.1–99.0)	0.89 (0.80–0.94)

## 4 Discussion

The clinical utility of the sFlt‐1/PlGF ratio for the prediction and diagnosis of PE has been extensively discussed in the literature [7, 8]. The ratio is a valuable tool for identifying pregnant women at high risk of developing PE [8]. Cases with an increased sFlt‐1/PlGF ratio strongly indicate an elevated risk of PE and adverse pregnancy outcomes, thus supporting clinical decisions toward intensive monitoring and potentially preterm delivery [9, 10]. The sFlt‐1/PlGF ratio has emerged as a practical biomarker for early diagnosis, significantly enhancing pregnancy management and helping prevent severe maternal and neonatal complications [11]. The clinical utility of this biomarker allows better identification of high‐risk pregnancies, even when measured between 24 to 28 weeks of gestation [12, 13]. Some studies highlight that these improvements in diagnostic decision‐making help reduce unnecessary hospitalizations for low‐risk women by improving diagnostic precision, thereby optimizing resource use [8, 10, 12].

This study presents a head‐to‐head comparison of the analytical and clinical performance of the MAGLUMI X3 (Snibe) and the Cobas e602 (Roche) for determining sFlt‐1, PlGF, and their ratio in a real‐world clinical setting. Our study provides novel evidence supporting the MAGLUMI X3 analyzer as a suitable alternative for laboratories currently using the Cobas e602. Until now, various analytical assays and platforms have been used to measure sFlt‐1 and PlGF concentrations based on published studies. Automated Roche systems were used in the majority of studies [7, 8, 9, 10, 11, 12, 13]. However, other analytical assays were tested in different studies. Some authors used ELISA assays; Nikui et al. used the ELISA, R&D System, Human VEGF R1/Flt‐1 Immunoassay, and Human PlGF Immunoassay [14]. Gannoun et al. used R&D Systems Quantikine ELISA Kits [15]. Bremner et al. used DELFIA Xpress sFlt‐1 and PlGF 1–2‐3 solid‐phase two‐site fluoroimmunometric assays using a DELFIA Xpress Random Access Immunoanalyzer and DELFIA Xpress instrument [16]. BRAHMS/Kryptor sFlt‐1/PlGF platform was used in the study of Andersen [17]. Simon et al. used both Elecsys immunoassay and BRAHMS sFlt‐1 Kryptor/BRAHMS PlGF plus Kryptor to compare both methods’ ratios on early diagnosis of PE and fetal growth restriction [18]. Similarly, Elecsys and Kryptor analyzers were compared in a study of Stepan et al. [19]. Zhu et al., in a relatively new study, used a chemiluminescence system CARIS 200 (Xiamen) [20]. The NICE document provides an overview of several tests recommended for use alongside standard clinical assessment, with specific cut‐off values provided for each test [21].

In our study, sFlt‐1 and PlGF measurements were strongly correlated between the two analyzers. However, Bland–Altman analysis demonstrated a statistically significant positive bias for sFlt‐1 on the MAGLUMI X3 analyzer compared with Roche, whereas PlGF values were comparable and showed no significant bias. As a result, the calculated sFlt‐1/PlGF ratios were lower on the Roche platform, which was reflected in the different platform‐specific clinical cut‐off values discussed below. Overall, the MAGLUMI X3 analyzer demonstrated solid analytical performance, supporting its reliability and suitability for routine clinical application.

Several studies investigate the diagnostic accuracy of specific cut‐off values of sFlt‐1, PlGF, and their ratio in the diagnosis of PE and other indications. Other studies suggest the ability of the sFlt‐1/PlGF ratio to distinguish PE from normal pregnancy in comparison with the control group [14]. Using a uniform cut‐off value for all gestational ages could lead to an inaccurate diagnosis. The study by Verlohen established and validated a set of gestational age‐specific cut‐off values for PE diagnosis [22]. However, the sFlt‐1/PlGF ratio has also been assessed in other settings. Dröge et al. found that the sFlt‐1/PlGF ratio was significantly higher in patients who developed adverse maternal or fetal outcomes compared to those who did not [9]. In summary, the sFlt‐1/PlGF ratio is a useful tool not only for the diagnosis and prediction of PE, but also for other diagnoses and clinical applications [23]. Nevertheless, the cut‐off values derived in our study differ from those commonly cited in the literature, primarily due to differences in study design. Unlike previous studies, our investigation did not include healthy controls and focused solely on a high‐risk population. Thus, the thresholds we established reflect optimal diagnostic performance specifically for our cohort and are not universally applicable without additional validation in broader populations.

In our study, regarding clinical insight, the performed tests did not show statistically significant differences when comparing the ROC curves of both tested methods for early and late PE. Similarly, Cohen's kappa indicated high agreement between the two methods in classifying patients for both early and late PE. Both compared methods demonstrated reasonable performance in terms of sensitivity, specificity, and accuracy, further supporting their comparable diagnostic value. As mentioned above, our study cohort included only the high‐risk population with PE in the differential diagnosis. Because the two platforms showed systematic differences in sFlt‐1 measurements, classification was based on platform‐specific cut‐off values derived separately for Roche and MAGLUMI X3. This finding aligns with the observed analytical bias, where the MAGLUMI X3 analyzer systematically yielded higher sFlt‐1 concentrations compared to Roche, resulting in a correspondingly higher sFlt‐1/PlGF ratio.

The key strengths of our study include its direct, parallel evaluation of the MAGLUMI X3 analyzer alongside the widely established Roche platform in an authentic clinical environment, involving a relevant high‐risk patient population. This rigorous approach provides robust evidence regarding the analytical performance and diagnostic reliability of the MAGLUMI X3 system. This evidence is particularly relevant for clinical laboratories considering the implementation of alternative analytical platforms, as it provides direct comparative data necessary for evidence‐based decision‐making.

A limitation of our study is that the relatively small sample size could influence the robustness of the findings. Healthy controls were not included in our study, in contrast to other studies. The clinical determination of PE was partly based on the methodology provided by Roche, meaning that the presence of PE could have been partially influenced by the methodology itself. However, the aim of the study was to compare the Snibe methodology with the routinely established Roche methodology, which is already deeply embedded in the routine diagnosis of PE. Another limitation is that the diagnosis was evaluated retrospectively; however, this approach reflects the real‐life design of the study.

## 5 Conclusion

The MAGLUMI X3 analyzer demonstrated acceptable analytical performance and diagnostic discrimination comparable to the routinely used Roche platform in sFlt‐1 and PlGF analyses. Despite systematic differences in sFlt‐1 values, a similar clinical classification was achieved when platform‐specific cut‐offs were applied. Further studies with larger patient cohorts and inclusion of healthy controls are warranted to refine these clinical thresholds and validate our preliminary findings.

## Author Contributions

P.B. – Writing original draft, statistical analysis, S.K. – Writing, analysis, conceptualization, Z.S. – Writing and clinical evaluation, D.R. – Statistical analysis, conceptualization, guarantor, J.R. – Conceptualization, guarantor, K.B. – sample collection and analysis, M.K. – clinical overview. All authors approved the final version and take full responsibility for the manuscript.

## Funding

This work was supported by the Ministry of Health, Czech Republic‐Conceptual Development of Research Organisation (FNPl‐00669806), 00669806; Cooperatio Programme, research area Medical Diagnostics and Basic Medical Sciences.

## Disclosure

The authors have nothing to report.

## Ethics Statement

The study was approved by the Ethics Committee of the University Hospital and Faculty of Medicine in Pilsen (approval No. 367/24) and conducted in accordance with the World Medical Association Declaration of Helsinki (2013 revision). Written informed consent was obtained from all participants prior to inclusion. Samples were analyzed on the MAGLUMI X3 according to laboratory standard operating procedures. Data handling complied with EU GDPR and local regulations, with restricted, audited access to study records.

## Consent

The authors have nothing to report.

## Conflicts of Interest

The authors declare no conflicts of interest.

## Supporting information


**Table S1:** 2 × 2 cross‐tabulations at proposed cut‐offs (with sensitivity and specificity, 95% CI).

## Data Availability

The data that support the findings of this study are available from the corresponding author upon reasonable request.
