# Supplementary material for: sFlt‐1/PlGF Ratio in the Diagnosis of Preeclampsia on the MAGLUMI X3 Analyzer
Source: J Clin Lab Anal. 2026 Jul 5:e70302. Online ahead of print. doi: 10.1002/jcla.70302 (PMC13399780; doi:10.1002/jcla.70302)
Supplement: Supplementary file 1 — Table S1: 2 × 2 cross‐tabulations at proposed cut‐offs (with sensitivity and specificity, 95% CI). [file JCLA-9999-e70302-s001.docx]

Supplementary Table S1. 2×2 cross-tabulations at proposed cut-offs (with sensitivity and specificity, 95% CI)

Diagnostic sensitivity and specificity with 95% confidence intervals.

# Early-onset PE — Roche (Elecsys®)

|  | PE present | PE absent |
| --- | --- | --- |
| Test positive | TP: 16 | FP: 6 |
| Test negative | FN: 2 | TN: 58 |

Sensitivity 72.7% (95% CI 49.8–89.3); Specificity 98.3% (95% CI 91.1–100.0)

# Early-onset PE — MAGLUMI® (Snibe)

|  | PE present | PE absent |
| --- | --- | --- |
| Test positive | TP: 16 | FP: 6 |
| Test negative | FN: 3 | TN: 57 |

Sensitivity 72.7% (95% CI 49.8–89.3); Specificity 95.0% (95% CI 86.1–99.0)

# Late-onset PE — Roche (Elecsys®)

|  | PE present | PE absent |
| --- | --- | --- |
| Test positive | TP: 14 | FP: 3 |
| Test negative | FN: 4 | TN: 43 |

Sensitivity 82.4% (95% CI 56.6–96.2); Specificity 94.0% (95% CI 83.5–98.7)

# Late-onset PE — MAGLUMI® (Snibe)

|  | PE present | PE absent |
| --- | --- | --- |
| Test positive | TP: 15 | FP: 2 |
| Test negative | FN: 6 | TN: 41 |

Sensitivity 88.2% (95% CI 63.6–98.5); Specificity 88.0% (95% CI 75.7–95.5)
